# Altitude‐Related Variation in Carbon, Nitrogen, and Phosphorus Contents and Their Stoichiometry of Woody Organs in the Subtropical Mountain Forests, South China

**DOI:** 10.1002/ece3.71451

**Published:** 2025-06-17

**Authors:** Chunlin Huo, Zhonghua Zhang, Gang Hu, Yinghua Luo

**Affiliations:** ^1^ Guangxi Key Laboratory of Forest Ecology and Conservation, College of Forestry Guangxi University Nanning China; ^2^ Key Laboratory of Environment Change and Resources Use in Beibu Gulf, Ministry of Education Nanning Normal University Nanning China; ^3^ Guangxi Key Laboratory of Earth Surface Processes and Intelligent Simulation Nanning Normal University Nanning China; ^4^ Laibin Jinxiu Dayaoshan Forest Ecosystem Observation and Research Station of Guangxi Laibin China

**Keywords:** altitude, Daming Mountain, ecological stoichiometry, phosphorus limitation, plant organs

## Abstract

Altitude‐induced variations in hydrothermal conditions and vegetation affect plant nutrients and induce tradeoffs in survival strategies. However, nutrient allocation to different plant organs along altitudinal gradients remains unclear. Here, 24 plots were established across eight altitudinal gradients (300, 500, 700, 900, 1100, 1200, 1300, and 1400 m) in subtropical forests on Daming Mountain, South China. We analyzed the altitudinal patterns and factors influencing carbon (C), nitrogen (N), and phosphorus (P) content and their ratios in the leaves, branches, and roots of woody plants. We found that branches had higher mean C content and C:N and C:P ratios than roots and leaves, leaves had higher N and P content than roots and branches, and roots exhibited a higher mean N:P ratio than the other organs. With increasing altitude, the leaf and branch C, C:N, and leaf C:P increased, whereas the leaf N and P, branch N and N:P, and root N:P decreased. Plant N:P ratios above 16 indicate that plant growth in the study area was mainly restricted by P. The positive correlation between N and P content across plant organs suggests synergistic absorption of these nutrients by plants. These results demonstrate that soil nutrients and stoichiometry directly influenced C, N, and P stoichiometry among different organs and that the soil C:P ratio was a common impact factor for these organs. These findings may elucidate the nutrient allocation patterns and adaptive strategies of plants in subtropical mountains and provide a foundation for forest management and restoration.

## Graphical abstract

In this study, we explore the altitudinal variations in stoichiometric traits of carbon (C), nitrogen (N), and phosphorus (P) across plant organs and identify the critical environmental factors affecting these traits in a subtropical evergreen broad‐leaved forest. These findings shed light on nutrient cycling, ecological strategies, and environmental adaptation mechanisms of subtropical forest plants, providing a foundation for forest management and restoration.

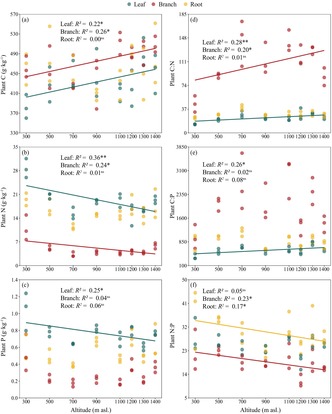

## 1 Introduction

Ecological stoichiometry is a critical method in modern ecological research, involving the examination of the biochemical constituents of organisms and energy equilibrium within ecosystems (Elser et al. 2000; Reich et al. 2006). Plant ecological stoichiometry explores elemental traits and their relationships with environmental factors and ecosystem processes (Elser et al. 2010; Sterner and Elser 2002). Leaves facilitate photosynthesis and respiration, branches facilitate nutrient transport, and roots play a pivotal role in nutrient uptake. The carbon (C), nitrogen (N), and phosphorus (P) content of these organs is closely linked to plant growth, nutrient efficiency, adaptation, and ecosystem nutrient cycling (Li, Fu, et al. 2023; Luo et al. 2020; Xing et al. 2022). Thus, investigating the stoichiometric traits of C, N, and P in plant leaves, branches, and roots is essential to elucidate plant resource use and allocation strategies.

Mountain ecosystems are characterized by an interplay between climate and topography, creating highly variable environments. Altitudinal gradients influence multiple environmental factors such as temperature, precipitation, and sunlight, which affect soil physicochemical properties, plant growth, and distribution (Bin et al. 2022; Körner 2007; Rahbek et al. 2019). Although numerous studies have focused on the response of leaf nutrients to altitude (Chen et al. 2022, 2024; Hong et al. 2024; Zhang et al. 2023), research on other plant organs, such as branches and roots, particularly in subtropical mountains, is limited. For instance, leaf N and P contents increased, and root N:P decreased with increasing altitude on Wuyi Mountain (Chen et al. 2020). Other patterns, such as an initial decrease followed by an increase, have also been observed (He et al. 2016; Liu et al. 2021; Zhu et al. 2024). These findings indicated that the altitudinal patterns of plant organs are ambiguous. Therefore, further research on nutrient allocation strategies and ecological adaptation mechanisms of plant organs at different altitudes in subtropical mountain ecosystems is of significant scientific value for understanding plant responses to environmental shifts.

The temperature‐plant physiology and temperature‐biogeochemistry hypotheses are key theories explaining the altitudinal impacts on plant ecological stoichiometry (Zhang et al. 2023). The former posits that plants increase N and P contents to compensate for reduced metabolic rates under low‐temperature conditions. This adaptation maintains organ growth and enhances resource competition and defense capabilities (Reich and Oleksyn 2004). Conversely, the latter suggests that low temperatures suppress soil microbial activity, which diminishes the availability of soil N and P, thereby leading to reduced N and P content in plants (Aerts and Chapin III 1999; Reich and Oleksyn 2004). Initially formulated for leaves (Zhang et al. 2023), the applicability of these hypotheses to other organs has not yet been comprehensively explored. A previous study on the Qinghai‐Tibet Plateau showed that the N and P contents in the leaves, stems, and branches increased with altitude, supporting the temperature‐plant physiology hypothesis (Zhang, Chen, et al. 2022). However, the opposite trend in the P content of these organs has been found in the Qilian Mountains, aligned with the temperature‐biogeochemistry hypothesis (Qin et al. 2022). Currently, there is a relative scarcity of research that applies these two hypotheses to the elemental stoichiometry of plant organs in subtropical regions. Further validation of these hypotheses across different plant organs in subtropical regions may provide deeper understanding of the spatial patterns of plant stoichiometric traits in subtropical ecosystems.

Daming mountain is one of the few remaining monsoon evergreen broadleaf forest areas near the Tropic of Cancer in China. It has well‐preserved vegetation and high plant diversity, making it one of the richest regions in South China in terms of plant resources. Its towering terrain exhibits distinct vertical variations in biota and climate (Li, Luo, et al. 2023; Li, Ye, et al. 2023). Previous studies have revealed changes in soil properties (Zhao et al. 2013) and plant community structures (Li, Luo, et al. 2023) with increasing altitude on this mountain. However, studies on plant organ stoichiometry, particularly on how C, N, and P contents and their ratios respond to changes in altitude in Daming mountain, have not been reported. In this study, based on altitudinal gradient plots, we analyzed the distribution of C, N, and P and their ratios in leaves, branches, and roots. This study aimed to (1) assess the altitudinal patterns of C, N, and P contents and their ratios in different plant organs and (2) identify key environmental factors affecting the C, N, and P stoichiometric traits of plant organs. These findings elucidate how plant organ C, N, and P contents adapt to altitudinal variations and their broader ecological implications, thereby offering a theoretical foundation for conserving plant diversity and multifunctionality in subtropical montane ecosystems.

## 2 Materials and Methods

### 2.1 Study Area

**FIGURE 1:**
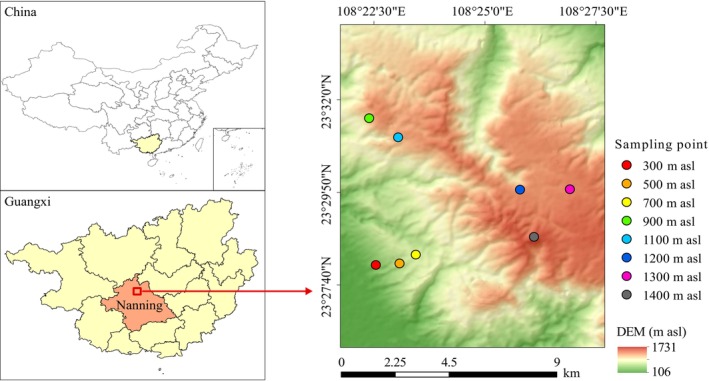
Location of forest plots along the altitudinal gradient of Daming mountain, South China.

The study area is situated in Daming mountain of Nanning City, Guangxi, South China (23°27′–23°32′ N, 108°22′–108°27′ E; Figure 1), which crosses the Tropic of Cancer and features a subtropical monsoon climate. It has a mean annual temperature of 15.1°C, with the highest temperatures reaching 21.9°C and the lowest dropping to 5.8°C. The region receives an average annual precipitation of 2630.3 mm, predominantly occurring between June and August, which constitutes approximately 50% of the annual precipitation. The area experiences an annual sunshine duration of 1295.4 and 1665.1 h and maintains an average annual relative humidity of 90%. Altitudes range from 115 to 1760 m, with soils primarily consisting of clay loam and loamy types, and soil pH ranging between 3.9 and 4.7 (Li, Luo, et al. 2023; Li, Ye, et al. 2023).

### 2.2 Sample Collection and Analysis

In July 2023, we selected eight altitudes between 300 and 1400 m for our study. At each altitude, three 20 × 20 m plots were established, spaced at least 100 m apart, totaling 24 plots. Geographic information such as longitude, latitude, altitude (Alt), slope degree, slope aspect, forest types, and dominant species was recorded (Table 1). Healthy leaves and branches of the dominant species were collected from different canopies and heights. Roots (diameter < 2 mm) and topsoil (0–20 cm) were collected from the four corners and diagonal center of each plot after the removal of surface debris. The leaf, branch, root, and soil samples were thoroughly mixed to form a composite sample for each plot, which yielded 96 samples. Plant samples in the laboratory were washed with clean water and dried to a constant weight in an oven at 70°C. The soil samples were dried naturally and debris was cleared. All samples were then passed through a 0.25‐mm mesh sieve.

**TABLE 1:** Basic information of eight altitudes.

Altitudes (m asl.)	Plot	Latitude	Longitude	Slope aspect	Slope degree (°)	Forest types	Dominant species
300	DMS01	23°28′03.35″	108°22′38.13″	South	19	Monsoon evergreen broadleaf forest	*Lindera metcalfiana*, *Microdesmis caseariifolia* and *Sloanea leptocarpa*
	DMS02	23°28′08.03″	108°22′32.70″	South	27		
	DMS03	23°28′02.10″	108°22′26.35″	Southeast	28		
500	DMS04	23°28′09.13″	108°23′05.95″	West	45	Monsoon evergreen broadleaf forest	*Clethra bodinieri*, *Engelhardia roxburghiana* and *Symplocos lancifolia*
	DMS05	23°28′10.14″	108°23′04.74″	South	25		
	DMS06	23°28′09.57″	108°23′01.75″	West	15		
700	DMS07	23°28′24.04″	108°23′27.16″	North	12	Montane evergreen broadleaf forest	*Rhodoleia championii*, *Rhododendron moulmainense* and *Clethra bodinieri*
	DMS08	23°28′22.50″	108°23′26.50″	Northwest	20		
	DMS09	23°28′23.28″	108°23′28.94″	North	18		
900	DMS10	23°31′35.70″	108°22′19.01″	West	40	Montane evergreen broadleaf forest	*Rhodoleia championii*, *Machilus thunbergii* and *Erythroxylum sinense*
	DMS11	23°31′34.18″	108°22′23.66″	Southwest	30		
	DMS12	23°31′29.54″	108°22′29.12″	Southwest	35		
1100	DMS13	23°31′07.96″	108°23′01.20″	Southeast	14	Montane evergreen broadleaf forest	*Castanopsis fargesii*, *Rhododendron cavaleriei* and *Morella rubra*
	DMS14	23°31′07.22″	108°23′02.64″	Southwest	19		
	DMS15	23°31′08.02″	108°23′07.01″	Southeast	20		
1200	DMS16	23°29′55.56″	108°25′46.66″	North	10	Montane evergreen deciduous mixed forest	*Castanopsis eyrei*, *Litsea elongata* and *Engelhardia roxburghiana*
	DMS17	23°29′53.80″	108°25′47.38″	Northwest	9		
	DMS18	23°29′58.75″	108°25′48.05″	North	16		
1300	DMS19	23°29′57.90″	108°26′53.92″	Northwest	3	Montane elfin forest	*Machilus thunbergii*, *Schima argentea* and *Engelhardia roxburghiana*
	DMS20	23°29′54.32″	108°26′54.89″	North	2		
	DMS21	23°29′53.69″	108°26′55.92″	North	2		
1400	DMS22	23°28′47.75″	108°26′05.63″	Southwest	8	Montane elfin forest	*Camellia fraterna*, *Michelia maudiae* and *Symplocos sumuntia*
	DMS23	23°28′47.47″	108°26′06.36″	Southwest	6		
	DMS24	23°28′48.06″	108°26′08.99″	Southwest	4		

Total plant C and soil organic C (SOC) were determined using the acidified dichromate (K_2_Cr_2_O_7_‐H_2_SO_4_) oxidation‐external heating method (Nelson and Sommers 1982). Plant and soil total N (STN) was measured using the micro‐Kjeldahl method (Bremner 1996). Plant and soil total P (STP) was extracted using the molybdenum‐antimony colorimetric method (Olsen and Sommers 1982). An electrode meter was used to measure the soil pH at a water: soil ratio of 2.5:1 (w:v). Soil water content (SWC) and bulk density were assessed using the drying and ring knife methods, respectively (Bao 2000). The soil properties at different altitudinal gradients are presented in Table 2.

**TABLE 2:** Characteristics of soil properties at different altitudinal gradients in the study area.

Soil properties	Altitudes (m asl.)							
	300	500	700	900	1100	1200	1300	1400
SOC (g·kg^−1^)	25.86 ± 4.29 a	43.82 ± 7.84 a	44.73 ± 9.21 a	45.14 ± 11.53 a	52 ± 17.76 a	61.62 ± 10.76 a	46.84 ± 3.64 a	36.96 ± 3.09 a
STN (g·kg^−1^)	2.28 ± 0.34 a	2.77 ± 0.23 a	3.01 ± 0.48 a	3.01 ± 0.71 a	2.71 ± 1.22 a	3.24 ± 0.15 a	2.81 ± 0.24 a	3.47 ± 0.79 a
STP (g·kg^−1^)	0.35 ± 0.05 bc	0.3 ± 0.02 c	0.3 ± 0.01 c	0.49 ± 0.11 ab	0.37 ± 0.05 bc	0.63 ± 0.13 a	0.24 ± 0.02 c	0.32 ± 0.04 bc
SCN	11.33 ± 0.95 a	15.65 ± 1.66 a	14.65 ± 0.71 a	14.87 ± 0.93 a	22.17 ± 6.48 a	18.8 ± 2.45 a	16.66 ± 0.18 a	11.52 ± 2.09 a
SCP	74.07 ± 10.02 a	146.35 ± 17.61 a	152.56 ± 31.42 a	103.82 ± 33.79 a	151.12 ± 57.51 a	106.34 ± 31.31 a	198.78 ± 18.28 a	117.3 ± 8.78 a
SNP	6.69 ± 1.14 a	9.35 ± 0.44 a	10.26 ± 1.71 a	6.8 ± 2.07 a	7.66 ± 3.75 a	5.54 ± 1.09 a	11.93 ± 1.07 a	10.76 ± 1.63 a
BD (g·cm^−3^)	0.89 ± 0.02 a	0.86 ± 0.07 a	0.87 ± 0.08 a	0.83 ± 0.07 a	0.71 ± 0.02 a	0.77 ± 0.04 a	0.69 ± 0.03 a	0.81 ± 0.04 a
SWC (%)	22.88 ± 1.24 d	40.82 ± 1.5 c	47.14 ± 3.06 bc	47.64 ± 3.36 bc	70.46 ± 3.34 a	68.41 ± 5.54 a	54.93 ± 5.47 b	75.62 ± 5.22 a
pH	3.42 ± 0.04 a	3.61 ± 0.04 a	3.69 ± 0.03 a	3.64 ± 0.08 a	3.51 ± 0.1 a	3.48 ± 0.22 a	3.67 ± 0.04 a	3.85 ± 0.05 a

*Note:* Values are means ± standard error (*n* = 3). Different litter letters indicate significant differences under different altitudes (*p* < 0.05).

Abbreviations: BD, bulk density; pH, soil pH; SCN, SCP, and SNP, soil C:N, C:P, and N:P ratios, respectively; SOC, soil organic carbon; STN, soil total nitrogen; STP, soil total phosphorus; SWC, soil water content.

### 2.3 Data Analysis

Before analysis, data normality and homogeneity of variance were tested using the Kolmogorov–Smirnov and Levene's tests, respectively. One‐way analysis of variance (ANOVA) coupled with post hoc Duncan's test was conducted to demonstrate significant differences in C, N, and P content and their ratios among plant organs and soil properties at different altitudes. Linear regression analysis was employed to evaluate the impact of altitude on C, N, and P content and their ratios across plant organs. Relationships between these variables were assessed using Pearson's correlation analysis. A redundancy analysis (RDA) was used to identify the primary factors affecting the stoichiometric traits of plant organs. To further explore the different pathways whereby altitude and soil properties mediate the response of stoichiometry in several plant organs, a piecewise structural equation model (SEM) was constructed. Owing to the strong correlations between variables within soil nutrients and stoichiometry, soil physicochemical properties, and the stoichiometry of plant organs, principal component analysis (PCA) was conducted, and the first principal component (PC1) was used to replace the variables (Table S1). Model fit was assessed using Fisher's C with *p*‐value > 0.05 and the Akaike information criterion (Grace 2006; Schermelleh‐Engel et al. 2003). One‐way ANOVA was conducted with SPSS 26.0 (IBM, Chicago, IL, USA), and regression, correlation, RDA, SEM, and PCA analyses were performed using the “stats,” “psych,” “vegan,” “piecewiseSEM,” and “FactoMineR” packages in R (R Core Team, version 4.3.1), respectively.

## 3 Results

### 3.1 Variations of Plant Organ C, N, and P Contents and Stoichiometry

Across all plants, the mean (±standard error) values of the leaf C, N, and P were 434.27 ± 8.49, 19.26 ± 0.89, and 0.77 ± 0.03 g·kg^−1^, respectively, and their ratios were 23.79 ± 1.28 for C:N, 586.55 ± 27.52 for C:P, and 25.14 ± 0.78 for N:P. For branches, the mean contents of C, N, and P were 476.19 ± 7.77, 5.10 ± 0.55, and 0.28 ± 0.03 g·kg^−1^, respectively, with ratios of C:N, C:P, and N:P at 108.55 ± 7.23, 1989.43 ± 159.63, and 18.64 ± 0.97, respectively. In roots, the corresponding stoichiometry was 455.88 ± 8.86, 15.94 ± 0.63, and 0.55 ± 0.03 g·kg^−1^ for C, N, and P, respectively. Their ratios were 29.39 ± 1.06, 874.68 ± 41.98, and 30.00 ± 1.35 for C:N, C:P, and N:P, respectively. The mean C, C:N, and C:P ratios were significantly higher in the branches than in the roots and leaves, whereas the mean N and P content was significantly higher in the leaves than in the roots and branches. Additionally, the roots displayed a significantly higher mean N:P ratio than the leaves and branches (*p* < 0.05, Table 3).

**TABLE 3:** C, N, and P contents and their ratios in plant organs.

Plant organs	C (g·kg^−1^)	N (g·kg^−1^)	P (g·kg^−1^)	C:N	C:P	N:P
Leaf	434.27 ± 8.49 b	19.26 ± 0.89 a	0.77 ± 0.03 a	23.79 ± 1.28 c	586.55 ± 27.52 c	25.14 ± 0.78 b
Branch	476.19 ± 7.77 a	5.10 ± 0.55 c	0.28 ± 0.03 c	108.55 ± 7.23 a	1989.43 ± 159.63 a	18.64 ± 0.97 c
Root	455.88 ± 8.86 ab	15.94 ± 0.63 b	0.55 ± 0.03 b	29.39 ± 1.06 b	874.68 ± 41.98 b	30.00 ± 1.35 a

*Note:* Values are means ± standard error (*n* = 3). Different litter letters indicate that significant differences among plant organs (*p* < 0.05).

### 3.2 Altitudinal Patterns of Plant Organ C, N, and P Contents and Stoichiometry

**FIGURE 2:**
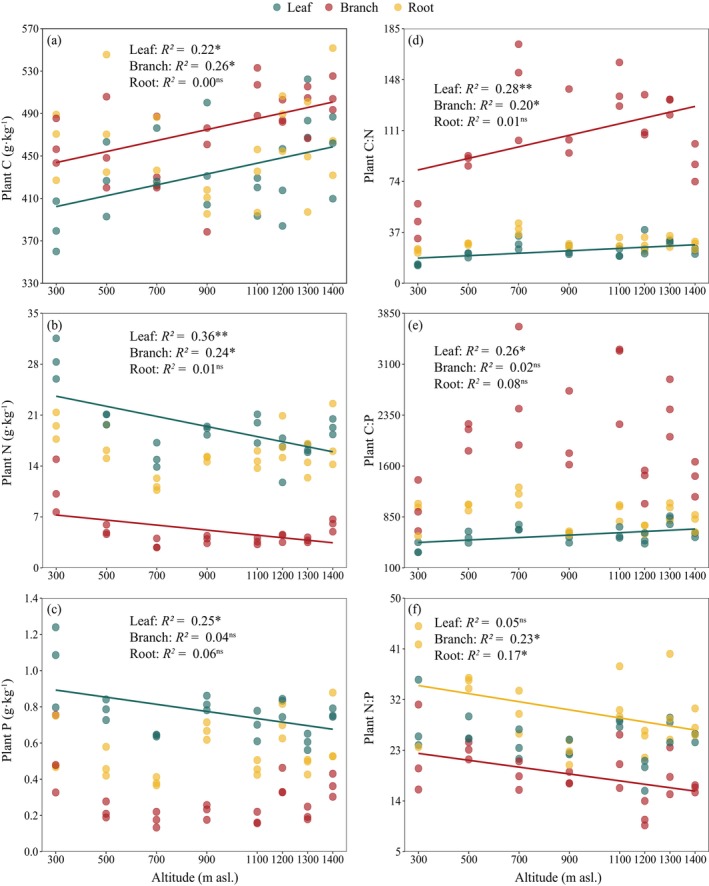
Variance of C, N, and P contents (a–c) and their ratios (d–f) among different organs along altitudes. ^ns^ *p* > 0.05, *p < 0.05, **p < 0.01.

With increasing altitude, leaf and branch C increased significantly, whereas leaf N, P, and branch N decreased significantly (*p* < 0.05); however, root C, N, P, and branch P showed no significant differences (Figure 2a–c). The leaf C:N and C:P ratios and branch C:N ratio increased with increasing altitude, whereas the branch and root N:P ratios decreased (*p* < 0.05). However, no significant differences were determined in the root C:N and C:P ratios, branch C:P ratios, or leaf N:P ratios (Figure 2d–f).

### 3.3 Correlation Between Plant Organ C, N, and P Contents and Stoichiometry

**FIGURE 3:**
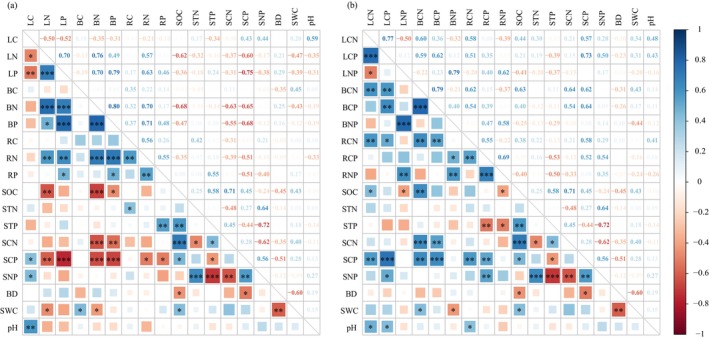
Correlation between C, N, and P contents (a) and their ratios (b) of different organs and soil properties. *p < 0.05, **p < 0.01, ***p < 0.001. BC, branch carbon; BCN, BCP, and BNP, branch C:N, C:P, and N:P ratios, respectively; BD, bulk density; BN, branch nitrogen; BP, branch phosphorus; LC, leaf carbon; LCN, LCP, and LNP, leaf C:N, C:P, and N:P ratios, respectively; LN, leaf nitrogen; LP, leaf phosphorus; pH, soil pH; RC, root carbon; RCN, RCP, and RNP, root C:N, C:P, and N:P ratios, respectively; SCN, SCP, and SNP, soil C:N, C:P, and N:P ratios, respectively; RN, root nitrogen; RP, root phosphorus; SOC, soil organic carbon; STN, soil total nitrogen; STP, soil total phosphorus; SWC, soil water content.

The N and P showed significant positive correlations across different plant organs, and a significant positive correlation was determined between N and P within the same organ. Leaf C was significantly negatively correlated with leaf N and P contents (*p* < 0.05, Figure 3a). The C:N and N:P ratios were positively correlated across different organs, whereas the C:P ratios of the leaves and branches were significantly positively correlated (*p* < 0.05, Figure 3b).

### 3.4 Effects of Environmental Factors on Plant Organ Stoichiometric Traits

SOC was significantly negatively correlated with leaf N, branch N, and P, and positively correlated with the C:N ratio of these organs. STN was significantly positively correlated with root C. STP was significantly positively correlated with root P and negatively correlated with root C:P and N:P ratios. Soil C:N (SCN) was significantly negatively correlated with branch N and P, but positively correlated with branch C:N and C:P. Soil C:P (SCP) demonstrated significant negative correlations with N and P content in plant organs, but positive correlations with the C:N and C:P ratios of these organs. Soil N:P (SNP) was significantly positively correlated with leaf C, C:P, and root C:P. SWC was significantly negatively correlated with leaf N, branch N, and N:P, and positively correlated with branch C and C:N. Soil pH was significantly positively correlated with leaf C, C:N, and C:P, along with root C:N (*p* < 0.05, Figure 3).

**FIGURE 4:**
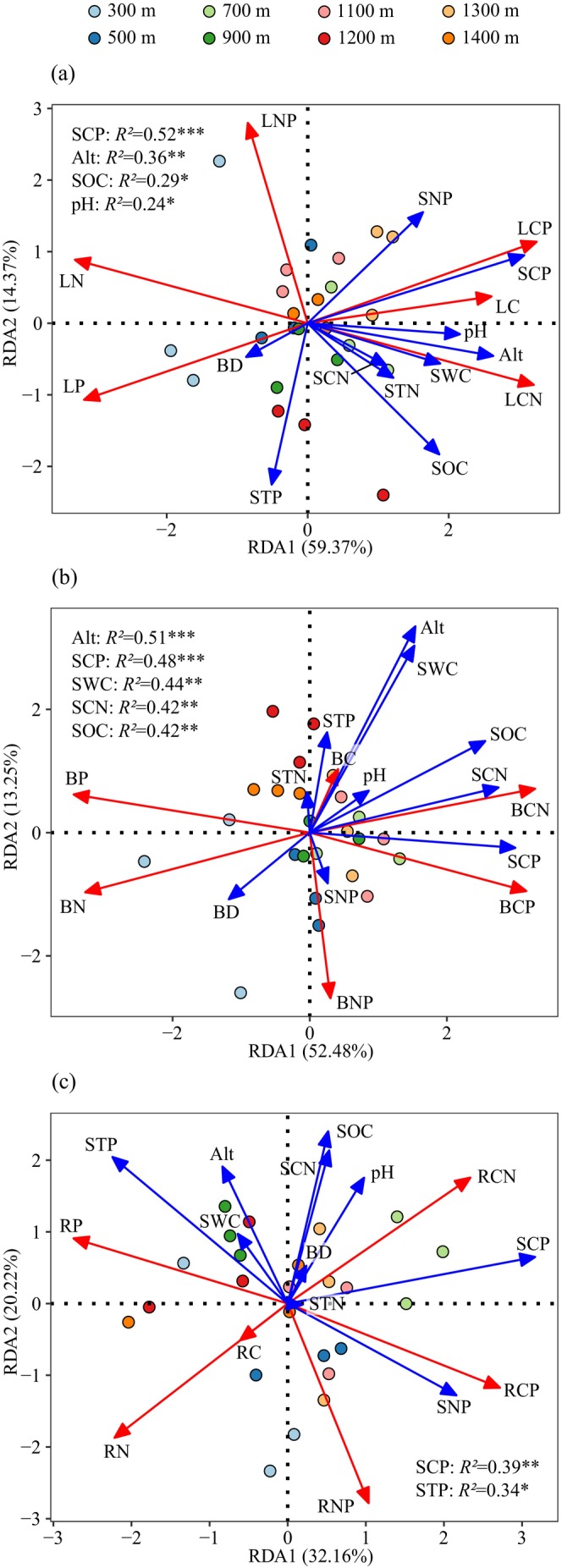
Redundancy analysis ordination for the leaf (a), branch (b), and root (c) stoichiometry and environmental factors. Red and blue arrows indicate response and explanatory variables, respectively. *R* ^*2*^ values indicate the proportion of variance explained. *p < 0.05, **p < 0.01, ***p < 0.001. Alt, altitude; BC, branch carbon; BCN, BCP and BNP, branch C:N, C:P and N:P ratios, respectively; BD, bulk density; BN, branch nitrogen; BP, branch phosphorus; LC, leaf carbon; LCN, LCP and LNP, leaf C:N, C:P, and N:P ratios, respectively; LN, leaf nitrogen; LP, leaf phosphorus; pH, soil pH; RC, root carbon; RCN, RCP, and RNP, root C:N, C:P, and N:P ratios, respectively; SCN, SCP, and SNP, soil C:N, C:P, and N:P ratios, respectively; RN, root nitrogen; RP, root phosphorus; SOC, soil organic carbon; STN, soil total nitrogen; STP, soil total phosphorus; SWC, soil water content.

The RDA results showed that RDA1 and RDA2 jointly accounted for 73.74%, 65.73%, and 52.38% of the variation in the stoichiometric traits of leaves, branches, and roots, respectively. SCP, Alt, SOC, and soil pH were the primary factors affecting the leaf stoichiometric traits (Figure 4a), whereas Alt, SCP, SWC, SCN, and SOC were the main determinants of the branch stoichiometric traits (Figure 4b). SCP and STP primarily influenced the root stoichiometric traits (Figure 4c).

**FIGURE 5:**
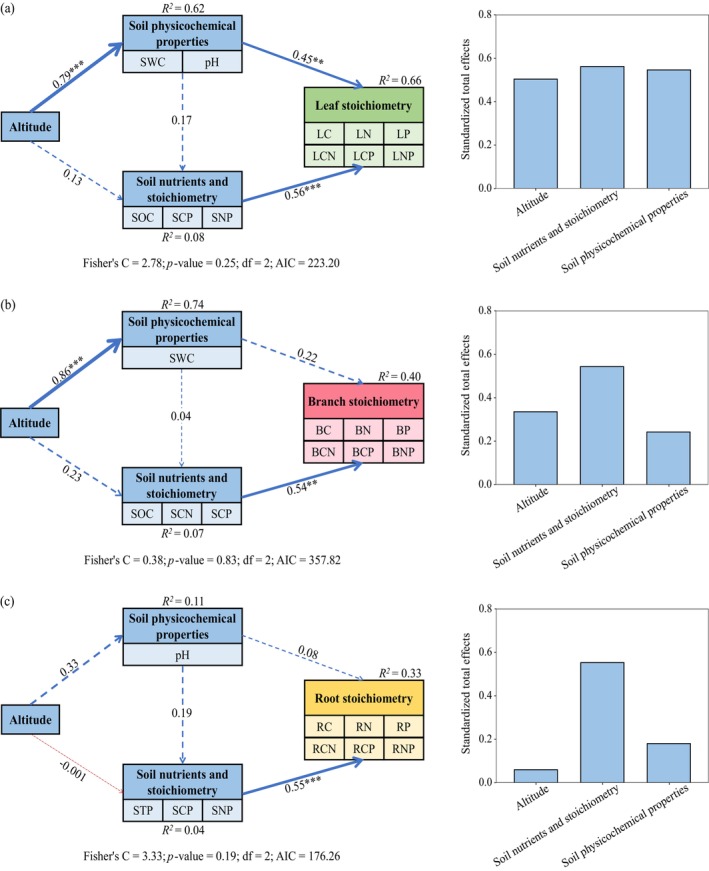
Structural equation model results for the pathways influencing the leaf (a), branch (b), and root (c) stoichiometry, and their standardized total effects. Numbers next to the arrows represent standardized path coefficients. Red and blue arrows denote negative and positive pathways, respectively. Solid and dashed arrows indicate significant and not significant pathways, respectively. The width of the arrows indicates the strength of the path coefficient. *R* ^*2*^ values indicate the proportion of variance explained. **p < 0.01, ***p < 0.001. BC, branch carbon; BCN, BCP, and BNP, branch C:N, C:P, and N:P ratios, respectively; BN, branch nitrogen; BP, branch phosphorus; LC, Leaf carbon; LCN, LCP, and LNP, leaf C:N, C:P, and N:P ratios, respectively; LN, leaf nitrogen; LP, leaf phosphorus; pH, soil pH; RC, root carbon; RCN, RCP, and RNP, root C:N, C:P, and N:P ratios, respectively; SCN, SCP, and SNP, soil C:N, C:P, and N:P ratios, respectively; RN, root nitrogen; RP, root phosphorus; SOC, soil organic carbon; STP, soil total phosphorus; SWC, soil water content.

Piecewise SEM results showed that altitude, soil nutrients and stoichiometry, and physicochemical properties accounted for 66%, 40%, and 33% of variation in leaf, branch, and root stoichiometries, respectively (Figure 5). Leaf stoichiometry was directly affected by soil nutrients and stoichiometry (SOC, SCP, and SNP). In addition, altitude had a significant indirect effect through soil physicochemical properties (SWC and pH; *p* < 0.05, Figure 5a). Soil nutrients and stoichiometry (SOC, SCN, and SCP) had a direct effect on branch stoichiometry (*p* < 0.05, Figure 5b). Soil nutrients and stoichiometry (STP, SCP, and SNP) also had a direct effect on root stoichiometry (*p* < 0.05, Figure 5c). More specifically, the total effects of altitude on leaf and branch stoichiometry were greater than those on root stoichiometry, and soil nutrients and stoichiometry had the greatest total effects on leaf, branch, and root stoichiometry compared to altitude and soil physicochemical properties.

## 4 Discussion

### 4.1 Patterns of C, N, and P Contents and Stoichiometry Among Plant Organs

Our study revealed that branches had the highest C, C:N, and C:P ratios and the highest N and P ratios, and roots had the highest N:P ratio (Table 3). These results are consistent with previous studies on terrestrial ecosystems (Tang et al. 2018) and southern subtropical regions (Zhang et al. 2024). Nutrient allocation is closely related to organ functional characteristics (Zhang, Zhao, et al. 2018). Branches, serving as structural and conductive organs, are rich in C‐containing compounds such as lignin and cellulose, which contribute to their high C, C:N, and C:P (Chen et al. 2018, 2021). Leaves require adequate N and P to synthesize the enzymes essential for photosynthesis and biochemical reactions, thereby leading to the highest levels of N and P (Tian et al. 2018). Roots that connect plants to the soil need to absorb a certain proportion of N and P to sustain life processes and support aboveground organs because of soil microbes and their own metabolic activities, resulting in a higher N:P ratio than that of leaves and branches (Chen et al. 2020; Li et al. 2021).

In our study, the leaf C (434.27 g·kg^−1^) and C:N (23.79, Table 3) were lower than those in Chinese forest (463.70 g·kg^−1^, 33.07) and subtropical China (475.17 g·kg^−1^, 32.59) (Chen et al. 2022; Tang et al. 2018). Lower C and C:N ratios are typically associated with higher specific leaf areas, photosynthesis, and growth rates (Niklas and Cobb 2005), suggesting that plants in our study area may have exhibited faster growth rates. The N:P ratio serves as a nutrient limitation indicator for plants, with N:P < 14 indicating N limitation, N:P > 16 denoting P limitation, and 14 < N:P < 16 signifying N and P co‐limitation (Aerts and Chapin III 1999; Koerselman and Meuleman 1996). The higher N:P ratios in leaves (25.14), branches (18.64), and roots (30.00, Table 3) may indicate that plants are relatively limited by P, which is consistent with findings in subtropical (Zhu et al. 2024) and tropical regions (Mo et al. 2019; van Breugel et al. 2019). This may be because the acidic soil in the study area is rich in iron and aluminum oxides, which strongly adsorb and fix P, leading to low available P (Johan et al. 2021; Zhao et al. 2013).

### 4.2 Response of the Plant Organ C, N, and P Contents and Stoichiometry to Altitude

In this study, a significant increase in leaf and branch C was observed with increasing altitude (Figure 2a), which may be attributed to plants accumulating more non‐structural C substances to enhance cellular osmotic pressure and cold resistance at high altitudes (Hoch and Körner 2012; Millard et al. 2007). In contrast, leaf N, P, and branch N decreased significantly (Figure 2b,c), in agreement with previous reports (Zhang et al. 2023; Zhao et al. 2014). This altitudinal tendency can be explained by the temperature‐biogeochemical hypothesis (Aerts and Chapin III 1999; Reich and Oleksyn 2004). As altitude increases, the cold environment limits microbial activity, thereby reducing the availability of soil N and P. Thus, leaf N, P, and branch N were lower in high‐altitude areas. We found that leaf N, P, and branch N decreased with altitude (Figure 2b,c), whereas STN and STP remained relatively stable (Table 2), likely because the availability of soil N and P may decrease with increasing altitude (Sundqvist et al. 2013; Vincent et al. 2014). Moreover, altitude significantly affected N and P in the leaves and branches, but soil nutrients (e.g., STN and STP) did not significantly influence these plant nutrients (Figure 3), likely because of the close relationship between leaf and branch nutrients as well as available soil nutrients (Bowman et al. 2003; Yin et al. 2021).

With increasing altitude, the leaf C:N, C:P, and branch C:N increased significantly, whereas the branch N:P decreased significantly (Figure 2d–f). Our results are consistent with those of Zhang et al. (2023) but in contrast with those of Zhang, Chen, et al. (2022) and Zhang, Feng, et al. (2022). The increase in leaf and branch C and decrease in leaf N, P, and branch N may explain the altitude‐related trends in leaf C:N, leaf C:P, and branch C:N. Fast‐growing plants typically exhibit low N:P and C:P ratios (Elser et al. 2003). In our study, habitat conditions were relatively better for plant growth in the lower altitude area, and the plants had a faster growth rate, thereby resulting in relatively low leaf C:N, C:P, and branch C:N. The decrease in branch N:P with altitude may be attributed to a reduction in branch N, whereas P in branches remained relatively stable.

### 4.3 Relationships Among C, N, and P Contents and Stoichiometry in Plant Organs

The significant positive correlations between N in leaves, branches, and roots as well as P in these organs (Figure 3a) highlight the synergistic utilization of the same nutrients by different plant organs (Zhang, He, et al. 2018). This characteristic is fundamental to plant biology and crucial for stable growth (Zheng and Shangguan 2007). The significant positive correlation between N and P in the leaves, branches, and roots (Figure 3a) likely stemmed from the high ATP demand for protein synthesis during growth. This correlation reflects the consistent variation in N and P in plants, where environmental conditions influencing P supply can affect N uptake, confirming the general pattern of positive N‐P correlations in higher plants (Sterner and Elser 2002; Wright et al. 2005). The negative correlations among leaf C, N, and P (Figure 3a) correspond to the principle that C is often negatively correlated with N and P in higher terrestrial plants (Sterner and Elser 2002), illustrating a trade‐off in nutrient allocation between structural development and growth (Zheng and Shangguan 2007). The C:N, C:P, and N:P ratios showed significant positive correlations across the leaves, branches, and roots (Figure 3b), demonstrating coordinated proportional nutrient uptake and utilization among plant organs. Furthermore, the absorption of one nutrient is closely linked to the supply of other nutrients, and any deficiency affects plant growth (Wang et al. 2020).

### 4.4 Factors Influencing Plant Organ Stoichiometric Traits

This study found that SCP was a common factor influencing the stoichiometric traits of the leaves, branches, and roots (Figures 4 and 5). SCP exhibited a significant negative correlation with P and a positive correlation with the C:P ratio in these organs (Figure 3), indicating that dynamic changes in plant organ stoichiometry are primarily influenced by P supply (Hedin 2004). As an indicator of soil nutrient constraints, SCP influences the efficiency of P absorption and transport in plants and C fixation, utilization, and N absorption. P is a key component of enzymes that are critical for photosynthesis, and its deficiency significantly reduces photosynthetic efficiency, affects C fixation and allocation, and limits N absorption (Hong et al. 2024; Wang et al. 2022). This explains why SCP was significantly negatively correlated with N in the leaves, branches, and roots and positively correlated with C:N in these organs (Figure 3). Moreover, the effects of SCP on leaf and root stoichiometric traits exceeded those of SOC and STP, respectively, and the influences of SCP and SCN on branch stoichiometric traits exceeded that of SOC (Figure 4), as confirmed by Bowman and Hurry (1993). Their research suggests that soil nutrient stoichiometry has a greater influence on leaf nutrients than on individual soil nutrients. This is because, in ecosystems with a low nutrient supply (such as the P‐limited ecosystem in our study area), plants tend to adopt conservative nutrient strategies, showing a minimal growth response to soil nutrient availability (Bowman and Hurry 1993). We found that SOC significantly affected the stoichiometric traits of leaves and branches, which were closely related to N and P, but weakly correlated with C in these organs (Figures 3 and 4). This may be because C is lost as CO_2_, whereas SOC primarily originates from microbially mediated amino acid metabolism (Delgado‐Baquerizo et al. 2015). In contrast, N and P return to their stable forms during nutrient cycling from plants to the soil (Zhang et al. 2019). STP was significantly correlated with root P, C:P, and N:P (Figure 3), possibly because plant roots secrete hydrolytic enzymes such as phosphatases to facilitate the mineralization of organic P (Rejmánková and Snyder 2008).

Altitude can alter temperature, water availability, light intensity, soil nutrients, and physicochemical properties, which may indirectly affect variations in plant nutrient content and ratios among organs (Zhu et al. 2024). Herein, altitude significantly influenced the stoichiometric traits of leaves and branches, but its effect on the roots was minimal (Figures 4 and 5) because the roots are mainly responsible for absorbing water and nutrients, and anchoring plants to the soil. To maintain these functions, the stoichiometric traits of roots may exhibit stronger homeostasis and a weaker response to external environmental changes (Jia et al. 2023). Therefore, roots may be less sensitive to variations in altitude than the above‐ground plant parts. The soil pH directly affects the availability of soil nutrients and plant nutrient uptake efficiency (Ding et al. 2022). These changes are rapidly reflected in the leaves through physiological processes, such as nutrient transport and allocation (He et al. 2015; Wang et al. 2022). In contrast, as the primary organ for nutrient absorption, root stoichiometric traits are directly influenced by local soil conditions. The effect of soil pH on root stoichiometry may be buffered or masked by other factors, such as root exudates and microbial activity (Liu et al. 2020; Tian et al. 2019). Therefore, soil pH was strongly related to leaf stoichiometry but had a minor effect on roots in our study (Figures 4 and 5). SWC significantly affected branch stoichiometric traits related to branch C, N, C:N, and N:P (Figures 3 and 4b). In addition to transporting water, branches are major growth sites pivotal for the development of new shoots and nutrient exchange (Ding et al. 2022; Zhang, Zhao, et al. 2018). SWC influences metabolic processes by altering cell membrane permeability and affecting the efficiency of nucleic acids and proteases, thereby affecting photosynthesis (Wang et al. 2023). Furthermore, research has indicated that climate, particularly precipitation and temperature, can directly or indirectly affect soil factors by improving plant–soil feedback responses (Luo et al. 2021; Xiong et al. 2024). Among these soil factors, soil nutrient availability directly reflects the changes in plant nutrient demands and nutrient content ratios among organs (Yin et al. 2021). Therefore, future studies should investigate how climatic factors and soil‐available nutrients affect C, N, and P stoichiometric traits in different plant organs.

## 5 Conclusions

This study revealed the altitudinal patterns of C, N, and P stoichiometry among different organs of woody plants and their responses to soil factors. Our results showed significant differences in C, N, and P contents and their ratios among different organs and significant correlations between N and P across these organs. Altitude had a significant effect on leaf and branch stoichiometry, but only a minor effect on roots. Specifically, leaf and branch N and leaf P decreased with increasing altitude, consistent with the temperature‐biogeochemistry hypothesis. The effects of soil nutrients and stoichiometry on leaf, branch, and root stoichiometry were greater than those of soil physicochemical properties, with SCP being a common driver of these organs. In addition, P was the limiting element for plant growth in the study area, suggesting that P fertilization could enhance soil effectiveness in forest management. These findings uncover the nutrient cycling, ecological strategies, and environmental adaptation mechanisms of subtropical forest plants.

## Author Contributions

**Chunlin Huo:** conceptualization (equal), data curation (equal), methodology (equal), writing – original draft (equal). **Zhonghua Zhang:** funding acquisition (equal), supervision (equal), writing – original draft (equal). **Gang Hu:** conceptualization (equal), funding acquisition (equal), supervision (equal), visualization (equal), writing – review and editing (equal). **Yinghua Luo:** project administration (equal), supervision (equal), visualization (equal), writing – review and editing (equal).

## Conflicts of Interest

The authors declare no conflicts of interest.

## Supporting information


Table S1

## Data Availability

The complete manuscript data can be accessed in Table S1.
