# Supplementary material for: Altitude‐Related Variation in Carbon, Nitrogen, and Phosphorus Contents and Their Stoichiometry of Woody Organs in the Subtropical Mountain Forests, South China
Source: Ecol Evol. 2025 Jun 17;15(6):e71451. doi: 10.1002/ece3.71451 (PMC12173710; doi:10.1002/ece3.71451)
Supplement: Supplementary file 1 — Table S1 [file ECE3-15-e71451-s001.docx]

**Supporting Information**

**Altitude-related variation in carbon, nitrogen, and phosphorus contents and their stoichiometry of woody organs in the subtropical mountain forests, south China**

Chunlin Huo^1^, Zhonghua Zhang^2,3^, Gang Hu^2,3^, Yinghua Luo^1,4^

^1^ Guangxi Key Laboratory of Forest Ecology and Conservation, College of Forestry, Guangxi University, Nanning, China

^2^ Key Laboratory of Environment Change and Resources Use in Beibu Gulf, Ministry of Education, Nanning Normal University, Nanning, China

^3^ Guangxi Key Laboratory of Earth Surface Processes and Intelligent Simulation, Nanning Normal University, Nanning, China

^4^ Laibin Jinxiu Dayaoshan Forest Ecosystem Observation and Research Station of Guangxi, Laibin, China

***Corresponding author**

Gang Hu, Key Laboratory of Environment Change and Resources Use in Beibu Gulf, Ministry of Education, Nanning Normal University, Nanning 530100, China; Email: hugang@nnnu.edu.cn

Yinghua Luo, Guangxi Key Laboratory of Forest Ecology and Conservation, College of Forestry,Guangxi University, Nanning 530004, China; Email: liliaceaeluo@163.com

**Supplementary Table:**

**Table S1** Results of the PCA of the stoichiometry of different organs (leaves, stems, and roots), soil nutrients and stoichiometry, and soil physicochemical properties for further piecewise structural equation models.

| a) Leaf stoichiometry | Response Factor | Predictor | PCA1 |
| --- | --- | --- | --- |
|  | Leaf stoichiometry | LC | 0.79*** |
|  |  | LN | -0.90*** |
|  |  | LP | -0.84*** |
|  |  | LCN | 0.94*** |
|  |  | LCP | 0.92*** |
|  |  | LNP | -0.26^ns^ |
|  |  | Cumulative (%) | 65.66 |
|  | Soil nutrients and stoichiometry | SOC | 0.44* |
|  |  | SCP | 0.96*** |
|  |  | SNP | 0.71*** |
|  |  | Cumulative (%) | 53.93 |
|  | Soil physicochemical properties | SWC | 0.76*** |
|  |  | pH | 0.76*** |
|  |  | Cumulative (%) | 57.49 |

| b) Branch stoichiometry | Response Factor | Predictor | PCA1 |
| --- | --- | --- | --- |
|  | Branch stoichiometry | BC | 0.12^ns^ |
|  |  | BN | -0.89*** |
|  |  | BP | -0.93*** |
|  |  | BCN | 0.92*** |
|  |  | BCP | 0.91*** |
|  |  | BNP | 0.09^ns^ |
|  |  | Cumulative (%) | 55.95 |
|  | Soil nutrients and stoichiometry | SOC | 0.91*** |
|  |  | SCN | 0.84*** |
|  |  | SCP | 0.66*** |
|  |  | Cumulative (%) | 66.17 |

| c) Root stoichiometry | Response Factor | Predictor | PCA1 |
| --- | --- | --- | --- |
|  | Root stoichiometry | RC | -0.24^ns^ |
|  |  | RN | -0.74*** |
|  |  | RP | -0.94*** |
|  |  | RCN | 0.75*** |
|  |  | RCP | 0.88*** |
|  |  | RNP | 0.37^ns^ |
|  |  | Cumulative (%) | 49.48 |
|  | Soil nutrients and stoichiometry | STP | -0.85*** |
|  |  | SCP | 0.78*** |
|  |  | SNP | 0.9*** |
|  |  | Cumulative (%) | 71.13 |

**Notes:** LC, leaf carbon; LN, leaf nitrogen; LP, leaf phosphorus; BC, branch carbon; BN, branch nitrogen; BP, branch phosphorus; RC, root carbon; RN, root nitrogen; RP, root phosphorus; SOC, soil organic carbon; STP, soil total phosphorus; LCN, LCP, and LNP, leaf C:N, C:P, and N:P ratios, respectively; BCN, BCP, and BNP, branch C:N, C:P, and N:P ratios, respectively; RCN, RCP, and RNP, root C:N, C:P, and N:P ratios, respectively; SCN, SCP, and SNP, soil C:N, C:P, and N:P ratios, respectively; SWC, soil water content; pH, soil pH. ^ns^ *p* > .05, * *p* < .05, *** *p* < .001.
